# Role of Keratinocytes in Sensitive Skin

**DOI:** 10.3389/fmed.2019.00108

**Published:** 2019-05-21

**Authors:** Matthieu Talagas, Laurent Misery

**Affiliations:** ^1^Laboratory on Interactions Neurons Keratinocytes (EA4685), Faculty of Medicine and Health Sciences, University of Western Brittany, Brest, France; ^2^Department of Pathology, Brest University Hospital, Brest, France; ^3^Department of Dermatology, Brest University Hospital, Brest, France

**Keywords:** sensitive skin, keratinocyte, sensory neuron, TRP, pain, itch

## Abstract

Sensitive skin is a clinical syndrome defined by the occurrence of unpleasant sensations such as burning, stinging, tingling, pricking, or itching in response to various normally innocuous physical, chemical, and thermal stimuli. These particular symptoms have led the consideration of a potential dysfunction of the intra-epidermal nerve fibers (IENF) that are responsible for pain, temperature, and itch perception. This neuronal hypothesis has just been reinforced by recent studies suggesting that sensitive skin could become assimilated to small fiber neuropathy. Meanwhile, the involvement of keratinocytes, the pre-dominant epidermal cell type, has so far mainly been considered because of their role in the epidermal barrier. However, keratinocytes also express diverse sensory receptors present on sensory neurons, such as receptors of the transient receptor potential (TRP) family, including Transient Receptor Potential Vallinoid 1 (TRPV1), one of the main transducers of painful heat which is also involved in itch transduction, and Transient Receptor Potential Vallinoid 4 (TRPV4) which is depicted as a heat sensor. While TRPV1 and TRPV4 are expressed both by sensory neurons and keratinocytes, it has recently been demonstrated that the specific and selective activation of TRPV1 on keratinocytes is sufficient to induce pain. Similarly, the targeted activation of keratinocyte-expressed TRPV4 elicits itch and the resulting scratching behavior. So, contrary to classical conception, the IENF are not the exclusive transducers of pain and itch. In light of these recent advances, this review proposes to consider the putative role of epidermal keratinocytes in the generation of the unpleasant sensations characteristic of sensitive skin syndrome.

## Introduction

**Figure 1:**
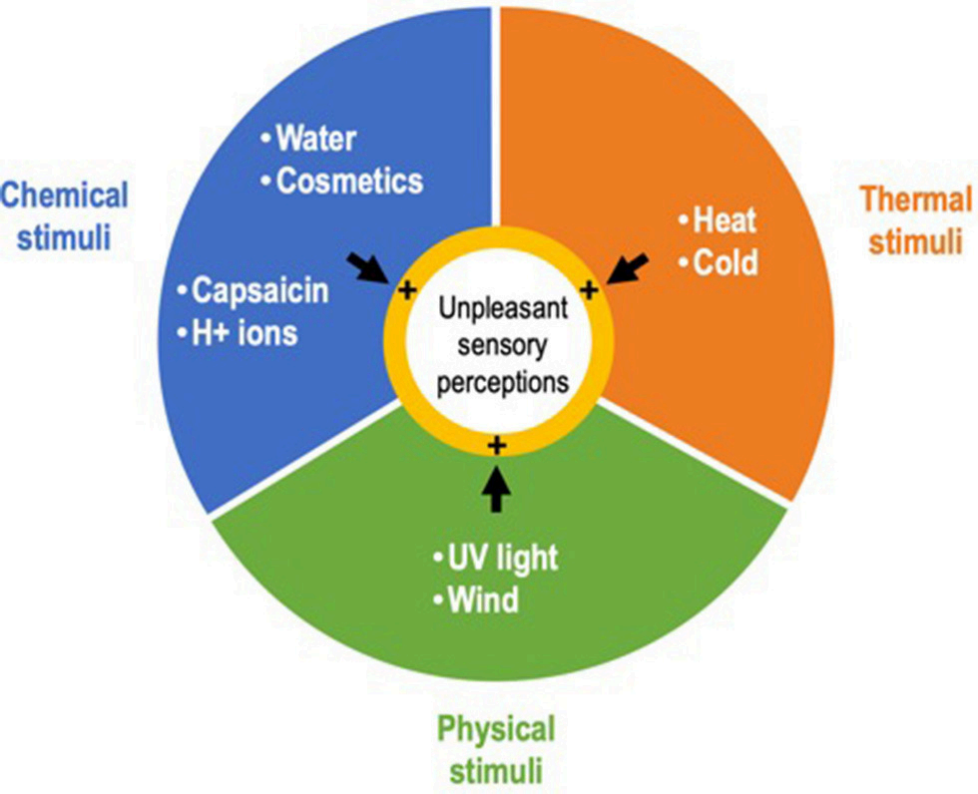
Main exogenous stimuli triggering the unpleasant sensory perceptions that characterize sensitive skin syndrome.

Sensitive skin syndrome (SSS) is a common skin condition (1) defined by the occurrence of unpleasant sensory perceptions such as burning, stinging, tingling, pricking, or itching, in response to various stimuli that normally should not provoke such sensations (2). These stimuli are mainly exogenous, physical (e.g., ultraviolet light, wind), thermal (heat, cold), and chemical (e.g., water, cosmetics, H^+^ ions, capsaicin) (Figure 1), but can also be endogenous, i.e., psychological or hormonal (3). The stinging test and capsaicin tests are frequently used to assess skin sensitivity in patients (4). An *in vitro* stinging test using lactic acid has also recently been proposed (5).

The pathophysiology of SSS has long been poorly understood. However, the presence of a wide variety of sensory symptoms related to pain and itch, and mostly triggered by environmental factors, suggested the implication of intra-epidermal nerve fibers (IENF) (6), conventionally described as the exclusive sensors for temperature, pain, and itch (7).

**Figure 2:**
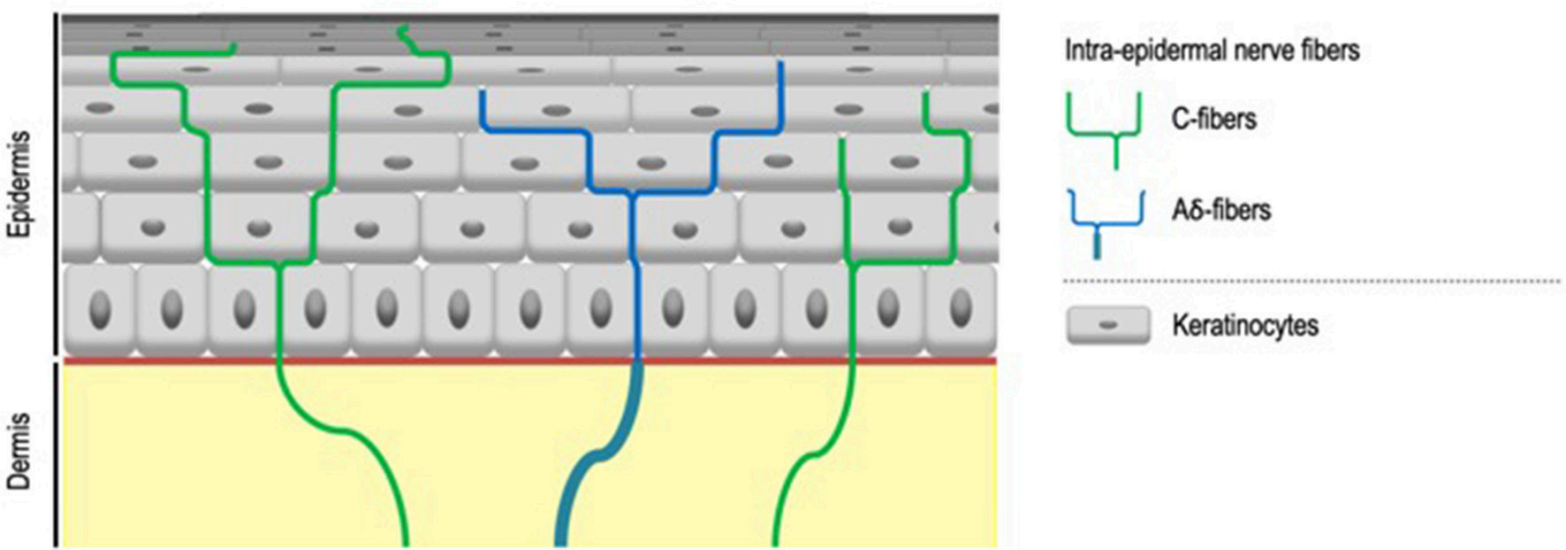
Intra-epidermal nerve fibers. Intra-epidermal nerve fibers, classified as C- and Aδ-fibers, unmyelinated, and thickly myelinated respectively, are conventionally described as the exclusive sensors for temperature, pain, and itch. Aδ-fibers lose their myelin sheath when crossing the basement membrane.

IENF, classified as C- and Aδ-fibers, unmyelinated, and thickly myelinated, respectively (7) (Figure 2), transduce stimuli via specific receptors, in particular Transient Receptor Potential (TRP) ion channels (8). TRP ion channels might therefore contribute to the abnormal sensations that characterize SSS especially since it turns out that they respond to a large range of physical, thermal and chemical stimuli also known to trigger sensory perceptions characteristic of SSS. First and foremost, TRP Vanilloid 1 (TRPV1), activated at temperatures above 42°C (9), but also by various stimuli such as capsaicin, low pH (9, 10), or ultraviolet (11), classically considered to be the main transducer of noxious heat (12) and also contributing to itch transduction (13), is proposed to play a role in the symptoms of SSS (6, 14, 15). Both up-regulation of cutaneous TRPV1 transcripts in sensitive skin compared to non-sensitive skin and the effectiveness of TRPV1 antagonist on SSS symptoms support this concept (16, 17).

Two main hypotheses, not inconsistent, have thus been proposed to explain the pathogenesis of SSS. According to the first one, SSS, considered as a consequence of dry skin, might result from an impaired epidermal barrier integrity leading to an insufficient protection of IENF. Therefore, numerous studies on alterations of the cutaneous barrier were performed (18). But a systematic review closed the debate by demonstrating that these abnormalities were not consistently found in subjects with sensitive skin (19), while sensitive skin may be associated with dry skin (a little more frequently), oily skin, mixed, or normal skin (from the point of view of cutaneous hydration) (20). The second hypothesis considers a neuronal dysfunction with a functional hyperactivity of sensory neurons (6, 14). Recent contributions suggesting that sensitive skin can be considered to be a small fiber neuropathy have just reinforced this neuronal hypothesis. First, using specific neuropathic pain questionnaires and quantitative sensory testing, clinical studies bring out a neuropathic component of pain in SSS (21). Second, SSS is associated with a decrease in PGP9.5 immunoreactive IENF density, arguing for nerve fiber alteration (22). The abnormal sensations would also be related to the hyperactivity and/or lowering of sensory thresholds in residual IENF. However, this discrepancy between the richness of sensory symptoms and the IENF density reduction may appear paradoxical (23). That goes without considering the involvement of keratinocytes, the pre-dominant epidermal cell type, whose role in SSS may go far beyond their simple contribution to the epidermal barrier. Indeed, recent studies have just demonstrated that they can also act as primary nociceptive transducers as a supplement to the sensory neurons. Hence, there is a need to consider the role of keratinocytes in sensitive skin pathophysiology from another perspective. In light of these recent results, this review considers the putative role of epidermal keratinocytes in contributing to the generation of unpleasant sensations characteristic of SSS.

## Prerequisite for Keratinocyte Contribution to Somatosensation

The skin is a sensory organ, by definition provided with a large range of sensory receptors. Except for IENF, all of them are classically described as being associated with non-neuronal component, whether Meissner corpuscles, Pacinian corpuscles, and Ruffini endings in the dermis or Merkel complexes in the epidermis (24).

Because of the apparent absence of specialized structures linking keratinocytes and IENF, epidermal keratinocytes were thus until recently considered to provide only physical support for IENF. It supported the idea that IENF were “free.” That goes without considering that, first, keratinocytes act as intermediaries between the environment and the IENF and are ideally located for perceiving exogenous stimuli. And second, the close contacts between keratinocytes and IENF (25–27) may be favorable for rapid and specific communication from keratinocytes to sensory neurons.

In addition, keratinocytes can release numerous substances capable of activating sensory neurons, such as glutamate (28) and acetylcholine (29) or ATP (30).

Furthermore, sensory receptors such as TRP ion channels are not only expressed by sensory neurons, but also by epidermal keratinocytes (31–33). Let's mention TRPV1 (34, 35), TRPV3 and TRPV4, which are mainly expressed by keratinocytes (36, 37), activated above 33 and 27°C (36, 38), and also described as transducing innocuous and noxious warmth (36, 38), or TRPM8 (39) and TRPA1 (40), both contributing to cold perception when expressed by sensory neurons (41–44). It has now been demonstrated that some of these TRP ion channels expressed by keratinocytes contribute to skin homeostasis in a thermo-dependent manner, helping to understand, for example, that the speed of skin barrier recovery is optimal between 36 and 40°C. Thus, TRPV1 activation either by capsaicin or by temperature delays barrier recovery following injury, whereas the TRPV1 antagonist capsazepin accelerates such recovery (45). In contrast, skin barrier recovery is accelerated by the activation of TRPV4 (45, 46).

## Keratinocytes can Transduce Nociceptive Responses

Beyond the contribution of keratinocyte-expressed TRP ion channels to skin homeostasis, the evidence of their ability to ultimately trigger pain or itch was until recently absent. Although it is essential to assert that epidermal keratinocytes can act as sensory cells in addition to sensory neurons, determining the respective contribution of epidermal keratinocytes and sensory neurons to sensory transduction remains a challenge. They are so closely associated that stimuli, whether chemical, thermal, or physical, affect both sensory neurons and keratinocytes. Therefore, studies in wild-type animals are useless. But this delicate obstacle has recently been overcome by using transgenic mouse models genetically configured to selectively express functional TRP ion channels in epidermal keratinocytes.

## Chemical and Thermal Stimuli

The proof that epidermal keratinocytes can transduce nociceptive and pruritic information in response to chemical stimuli has just been supplied by recent *in vivo* studies allowing the selective activation of TRPV1 or TRPV4 in keratinocytes without simultaneously stimulating adjacent IEFN.

**Figure 3:**
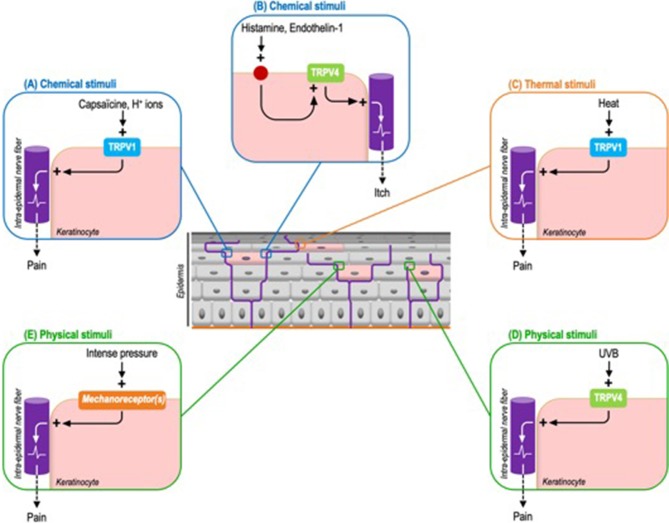
Identified exogenous stimuli and the corresponding keratinocyte sensory receptors triggering pain or itch in mouse models. **(A)** Chemical stimuli. Capsaicin can induce pain through the activation of keratinocyte-expressed TRPV1. By extension this process probably concerns H+ ions. **(B)** Chemical stimuli. Histamine and endothelin-1 activate TRPV4 through their respective receptors. Downstream TRPV4 activation induces itch. **(C)** Thermal stimuli. TRPV1 is also the main transducer of noxious heat. **(D)** Physical stimuli. UVB activate keratinocyte-expressed TRPV4 to induce pain. **(E)** Like intense pressure via not yet identified mechanoreceptor(s).

Thus, while TRPV1 is expressed both by sensory neurons and keratinocytes, its specific and selective activation on keratinocytes is sufficient to induce pain. Cutaneous applications of capsaicin in *Trpv1* global knockout mice genetically engineered to exclusively express TRPV1 in epidermal keratinocytes, via the CK5 promoter, is sufficient to elicit nocifensive behaviors and to induce the expression of c-fos, a neuronal activation marker, in the ipsilateral dorsal horn of the spinal cord (47). As TRPV1 expression is restricted to keratinocytes, this model demonstrates that keratinocytes are capable of transducing chemical nociceptive information via TRPV1, in addition to adjacent sensory neurons. TRPV1 being a polymodal receptor also activated by H^+^ ions and noxious heat, one may reasonably advance that other chemical stimuli, like H^+^ ions, and noxious warmth also probably elicit a perception of pain through the keratinocyte transduction (Figures 3A,C).

Based on a comparable strategy, by using mice genetically configured to induce a keratinocyte-specific tamoxifen-inductible *Trpv4* knockout, the pro-pruritic role of keratinocyte-expressed TRPV4 has also newly been demonstrated. The scratching behavior triggered by the intra-dermal injection of histaminergic pruritogens, like histamine and endothelin-1, is significantly reduced in keratinocyte *Trpv4* knock-down mice compared to wild-type mice. Similar results are obtained with selective inhibitors of TRPV4 but not with non-histaminergic compounds like chloroquine. Next, the activation of TRPV4 leads to a cytoplasmic calcium influx and the phosphorylation of the mitogen-activated protein kinase ERK, whose inhibition also reduces the perception of itch (48). Interestingly, this study uncovers that TRPV4 functions downstream of histaminergic itch receptors. As observed in sensory neurons, TRPV4 is coupled to pruritogen receptors, possibly to amplify the intra-keratinocyte signal and therefore optimize sensory perception (Figure 3B). Beyond these chemical stimuli, the contribution of warm temperatures to itch perception via keratinocyte-expressed TRPV4 remains to be determined.

The participation of TRPV4 in serotonin-mediated itch has also been reported in a *Trpv4* global knockout (49). But although TRPV4 is mainly expressed by keratinocytes compared to sensory neurons (36), the contribution of epidermal keratinocytes cannot be evaluated. Additional keratinocyte selective knockouts would be needed to assert that a coupling between keratinocyte-expressed TRPV4 and serotoninergic receptors induces itch-related scratching behaviors.

## Physical Stimuli

Using an optogenetic mouse model expressing channel-rhodopsin (ChR2), a blue-light-gated cation channel, exclusively in epidermal keratinocytes, Baumbauer et al. have also demonstrated that skin exposure blue-light is sufficient to induce action potentials in Aδ- and C-fibers and *in fine* trigger nocifensive behaviors. Conversely, optogenetically silencing keratinocytes through the expression of halo-rhodopsin reduces the activation of IENF (50). Moreover, this elegant demonstration reveals that the light stimulation of keratinocytes activates many subtypes of sensory neurons identified as responding to mechanical and/or thermal stimuli. In accordance with the previous data based on the keratinocyte expression of TRPV ion channels, these observations underscore the indisputable implication of epidermal keratinocytes in cutaneous sensory transduction.

In line with these results, evidence of the capacity of epidermal keratinocytes to transduce noxious mechanical stimuli has also been recently provided. Indeed, cutaneous light stimulation of mice expressing archae-rhodopsin-3 (Arch), a cation channel inducing cell membrane hyperpolarization, in epidermal keratinocytes, decreases the activation of C-fibers and inhibits mice's responses to noxious mechanical stimuli (Figure 3E). And more specifically, mechanically-stimulated keratinocytes activate sensory neurons through the release of ATP (51). Interestingly, these optogenetic mouse models appear complementary to those based on keratinocyte selective expression of TRP ion channels. Through the use of ectopic light-activated channels that modulate cell membrane voltage, they allow to overcome the fact that some receptors, such as keratinocyte mechanoreceptors, have not yet been identified.

In addition to the TRPV4 contribution to itch transduction described above, recent data also indicate that TRPV4 expressed by keratinocytes responds to acute UVB exposure by triggering allodynia (Figure 3D). Thus the keratinocyte-specific *Trpv4* knockout is associated with attenuated responses to noxious thermal and mechanical stimuli compared to wild type mice. Moreover, keratinocyte-expressed TRPV4 induces epidermal injury via the release of endothelin-1, a pro-nociceptive and pruriceptive substance, in response to UVB radiations. Downstream, endothelin-1 potentiates the pro-algesic action of keratinocyte-expressed TRPV4 via endothelin receptors in an autocrine and paracrine pathway. Consistent with these observations, TRPV4 and endothelin-1 immunoreactivities are increased in human skin following UVB exposure. This helps explain why sunburn and tissue damage are reduced in mice devoid of TRPV4 exclusively in epidermal keratinocytes. Furthermore, a *Trpv4* global knockout is not associated with an additional reduction in damage, underlining the pivotal contribution of keratinocytes (52).

## Conclusion and Perspectives

**Figure 4:**
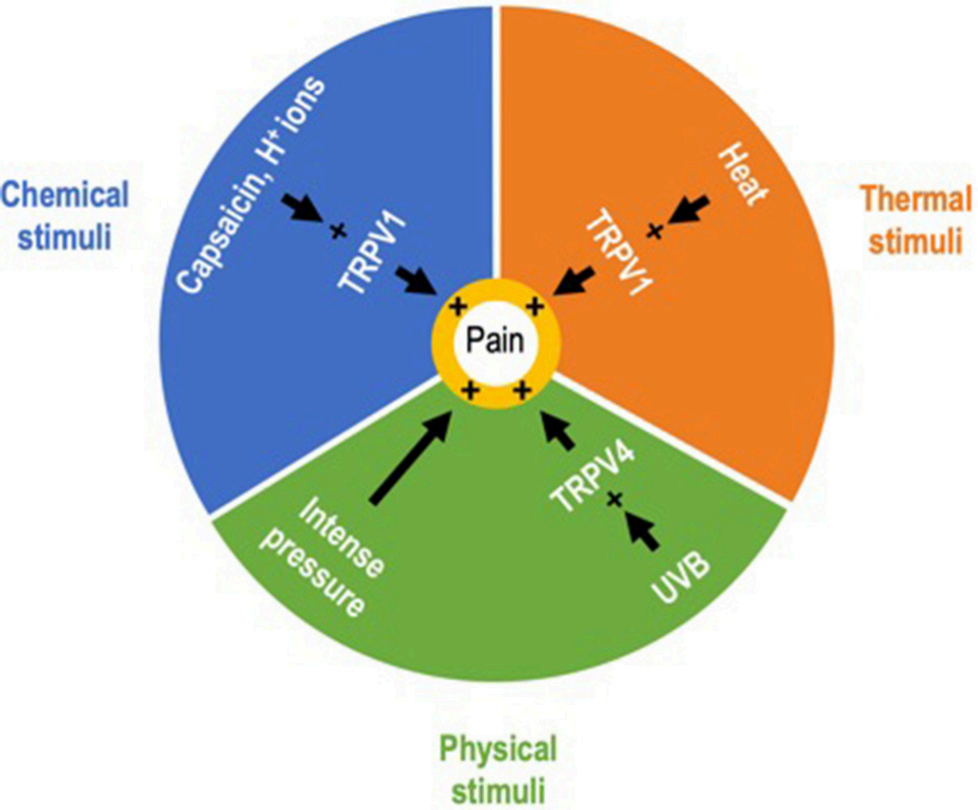
Exogenous painful stimuli. Correspondence between keratinocytes and sensitive skin syndrome. Epidermal keratinocytes can transduce nociceptive information from exogenous stimuli belonging to the three categories of stimuli identified as provoking the unpleasant sensory perceptions characteristic of sensitive skin syndrome.

Hence, the role of keratinocytes in sensitive skin may not be limited to alterations of the epidermal barrier but could also be related to their sensory properties. The identification of epidermal keratinocytes as primary sensory transducers is a paradigm shift in the field of cutaneous sensory perception. Contrary to classical conception, the IENF are not the exclusive transducers of pain and itch (53). These findings regarding keratinocytes demonstrate an expanded role for epithelial cells, and beyond them of the entire epidermis that may be considered as a sensory epithelium (54). Provided that epidermal keratinocytes contribute to abnormal sensations in SSS, the words “sensitive skin” would take on their full meaning. Indeed, it is remarkable to note that epidermal keratinocytes can transduce painful information from all categories of exogenous stimuli known to provoke unpleasant sensory sensations characteristic of SSS (Figure 4). Further studies on TRP ion channels in keratinocytes may help explain the pathophysiology of SSS and bring about changes in the management of SSS. If this assumption is confirmed, the characterization of the mechanism underlying communication between keratinocytes and sensory neurons will constitute another step in uncovering this newly identified cutaneous two-site receptor (55). But understanding the interactions between keratinocytes and sensory neurons remains a challenge as ambitious as it was to demonstrate the sensory role of keratinocytes. In addition to transgenic mouse models allowing the selective expression or suppression of keratinocyte receptors, keratinocyte-sensory neuron co-culture models that allow selective stimulation of keratinocytes and physically separate the two cell populations may be a useful tool to progress in this way (56). Both keratinocytes and the mechanisms by which keratinocytes activate sensory neurons maybe potential new therapeutic targets for SSS.

## Data Availability

The datasets generated for this study are available on request to the corresponding author.

## Author Contributions

MT wrote the manuscript. LM participated in the manuscript writing.

### Conflict of Interest Statement

The authors declare that the research was conducted in the absence of any commercial or financial relationships that could be construed as a potential conflict of interest.
